# [^18^F]FET PET-Guided management of pseudoprogression in glioblastoma (FET POPPING): the study protocol for a diagnostic randomized clinical trial

**DOI:** 10.1186/s13063-025-08921-8

**Published:** 2025-06-17

**Authors:** V.J. Ruijters, T.J. Snijders, J.A.J. van der Pol, E.M. van de Giessen, J.M. Niers, M.P.G. Broen, M.M. Anten, S.E.M. Veldhuijzen van Zanten, M. Geurts, A.I.J. Arens, D.J.H.A. Henssen, J.M. Gijtenbeek, M. van der Meulen, A.E.J. Sijben, E. Ghariq, M.J. Vos, J. Tim, I.B. Bosma, G.N. Stormezand, G.W.J. Frederix, J.W. Dankbaar, P.A. Robe, J.J.C. Verhoeff, F.Y.F.L. de Vos, M.G.E.H. Lam, R.M.T. ten Ham, N. Tolboom

**Affiliations:** 1 Department of Neurology (VJR, TJS), Department of Epidemiology and Health Economics, Julius Centre (GWJF, RMTtH), Radiology (JWD), Neurosurgery (PAR), Medical Oncology (FYFLdV) and Nuclear Medicine (MGEHL, NT), UMC Utrecht, Utrecht, Netherlands; 2 Department of Neurology (MPGB, MMA) and Nuclear Medicine (JAJvdP), Maastricht UMC, Maastricht, Netherlands; 3 Department of Radiology and Nuclear Medicine (EMvdG), Neurology (JMN) and Radiation Oncology (JJCV), Cancer Center Amsterdam, Amsterdam UMC, Amsterdam, Netherlands; 4 Department of Radiology and Nuclear Medicine (SEMVvZ) and Neurology (MG), Erasmus MC, Rotterdam, Netherlands; 5 Department of Radiology and Nuclear Medicine (AIJA, DJHAH) and Neurology (JMG), Radboud UMC, Nijmegen, Netherlands; 6 Department of Neurology (MvdM, AEJS) and Radiology and Nuclear Medicine (EG), Medisch Spectrum Twente, Enschede, Netherlands; 7 Department of Neurology (MJV) and Radiology and Nuclear Medicine (JT), Haaglanden MC, The Hague, Netherlands; 8 Department of Neurology (IBB) and Nuclear Medicine (GNS), UMC Groningen, Groningen, Netherlands

**Keywords:** Glioblastoma, GBM, Nuclear medicine, Neuro-oncology, [^18^F]FET PET, Tumor progression, Pseudoprogression, Radionecrosis, Unnecessary interventions, Health-related quality of life

## Abstract

**Background:**

During follow-up of glioblastoma patients after chemoradiation, expert teams often observe abnormalities on MRI with difficulty in distinguishing between tumor growth and pseudoprogression. Although advanced MRI techniques such as perfusion weighted imaging provide additional information, diagnostic uncertainty often remains, leading to incorrect or delayed diagnosis, and inappropriate treatment, such as unnecessary surgery. [^18^F]Fluoro-ethyl-tyrosine positron emission tomography (FET PET) has good discriminating power for this setting. Still, this diagnostic tool is not used frequently in The Netherlands due to costs, logistics and uncertainty about clinical benefit. In the FET POPPING study, we aim to determine the added value of [^18^F]FET PET for clinical management of glioblastoma patients.

**Methods:**

A multicenter diagnostic randomized clinical trial will be performed, from August 2024 until December 2027. Adult patients (*n*=144) with isocitrate dehydrogenase (IDH)-wildtype glioblastoma will be included, who, at least ≥3 months after the concomitant phase of standard temozolomide-based chemoradiation, have new or increased contrast enhancement on MRI, causing doubt between tumor growth or pseudoprogression.

In this trial, pseudoprogression will be used as an encompassing term that includes radionecrosis and other treatment-related changes after (chemo-)radiotherapy. Included patients will be randomized 1:1 in two arms. The investigational arm receives an additional [^18^F]FET PET scan, and clinical management is based on the index MRI and [^18^F]FET PET together. Clinical management of the control arm is based on the index MRI alone. Exact clinical management, as based on the available imaging, is chosen at the discretion of the local multidisciplinary board. The primary study endpoints are (a) the percentage of patients undergoing unnecessary interventions and (b) health-related quality of life after 12 weeks. Secondary endpoints include time-to-diagnosis, overall survival, and cost-effectiveness.

**Discussion:**

We hypothesize that the clinical management guided by an additional [^18^F]FET PET scan leads to fewer unnecessary interventions, better health-related quality of life after 12 weeks and among others reduced net healthcare costs, compared with management based on MRI only.

**Trial registration:**

The trial is registered on ClinicalTrials.gov on the 24th of June 2024, with registration number NCT06480721.

## Administrative information

**Table:** 

Title {1}	FET PET-Guided management of pseudoprogression in Glioblastoma (FET POPPING): the study protocol for a diagnostic randomized clinical trial
Trial registration {2a and 2b}	The trial is registered on ClinicalTrials.gov on the 24th of June 2024, with registration number NCT06480721
Protocol version {3}	Date 16–05–2025, Version 4.0
Funding {4}	ZonMw, Curium™ (unrestricted educational grant)
Author details {5a}	^1^ Department of Neurology (VJR, TJS), Department of Epidemiology & Health Economics, Julius Centre (GWJF, RMTtH), Radiology (JWD), Neurosurgery (PAR), Medical Oncology (FYFLdV) and Nuclear Medicine (MGEHL, NT), UMC Utrecht, Utrecht, Netherlands ^2^ Department of Neurology (MPGB, MMA) and Nuclear Medicine (JAJvdP), Maastricht UMC, Maastricht, Netherlands ^3^ Department of Radiology & Nuclear Medicine (EMvdG), Neurology (JMN) and Radiation Oncology (JJCV), Cancer Center Amsterdam, Amsterdam UMC, Amsterdam, Netherlands ^4^ Department of Radiology & Nuclear Medicine (SEMVvZ) and Neurology (MG), Erasmus MC, Rotterdam, Netherlands ^5^ Department of Radiology & Nuclear Medicine (AIJA, DJHAH) and Neurology (JMG), Radboud UMC, Nijmegen, Netherlands ^6^ Department of Neurology (MvdM, AEJS) and Radiology & Nuclear Medicine (EG), Medisch Spectrum Twente, Enschede, Netherlands ^7^ Department of Neurology (MJV) and Radiology & Nuclear Medicine (JT), Haaglanden MC, The Hague, Netherlands ^8^ Department of Neurology (IBB) and Nuclear Medicine (GNS), UMC Groningen, Groningen, Netherlands
Name and contact information for the trial sponsor {5b}	UMC Utrecht Dr. N. Tolboom, MD PhD Nuclear medicine physician UMC Utrecht 3508 GA Utrecht N.tolboom@umcutrecht.nl
Role of sponsor {5c}	UMC Utrecht has the leading role in the design, collection, management, analysis, interpretation of data, writing of the report and the decision to submit Curium™ does not have a role in the design, execution, analysis, manuscript writing and/or decision to publish

## Introduction

### Background and rationale {6a}

Glioblastomas are incurable progressive brain tumors with a variable, but poor prognosis. After surgery, chemoradiation with concomitant and adjuvant temozolomide chemotherapy offers effective, albeit temporary, palliation for many patients, with median overall survival of 14–16 months [1, 2]. In the months following radiotherapy, ~ 50% of patients will develop changes on MRI, suggestive of recurrence. In a majority of these patients, these MRI changes are classified as pseudoprogression [3]. Pseudoprogression is defined as treatment-induced increasing, or new, contrast enhancement which is mostly self-limiting and requires a different management strategy than tumor recurrence. Pseudoprogression will be used in this paper as an encompassing term that includes radionecrosis and other treatment-related effects after (chemo-)radiotherapy. Discrimination between pseudoprogression and tumor progression on MRI can be notoriously challenging [4]. In the patients that are eventually diagnosed with pseudoprogression, initial diagnostic uncertainty is common, which may lead to an increased risk of misdiagnoses and unnecessary interventions [5, 6].

Currently, in the Netherlands, in these patients with diagnostic uncertainty on MRI, the multidisciplinary team prefers to wait for a next scan in 6–12 weeks’ time for definitive diagnosis, leading to potential delay of diagnosis and adequate management and causing psychological burden for patients and their relatives with impact on quality of life [7].

The use of positron emission tomography (PET) with the radioligand [^18^F]Fluoro-ethyl-tyrosine, or [^18^F]FET PET in short, has the potential to differentiate treatment-related changes from tumor recurrence with higher accuracy compared to current clinical standard MRI including perfusion-weighted MRI [8–10]. Meta-analyses of literature confirm that addition of [^18^F]FET PET to MRI has higher diagnostic accuracy for differentiation of pseudoprogression from tumor recurrence compared with MRI alone [11, 12]. Recent literature has led to evidence-based recommendations by the Response Assessment in Neuro-Oncology (RANO) working group to add [^18^F]FET PET imaging to MRI in cases with diagnostic uncertainty [13].

However, this advice is not based on prospective randomized patient benefit- or cost-effectiveness studies, as neither have been performed.

Many studies of diagnostic test accuracy are available, but demonstrating high diagnostic accuracy is not equivalent to proving added value in clinical practice; since a diagnostic study is only the starting point of a clinical trajectory, clinical utility of a diagnostic test is best demonstrated in the context of the appropriate clinical workflow. A second problem for implementation of [^18^F]FET PET is that imaging costs are perceived to be relatively high, at least higher than an MRI with perfusion and in the light of the expected prognosis [14]. In somewhat comparable clinical settings, studies demonstrate that the addition of [^18^F]FET PET imaging is cost-effective [15, 16]. However, no cost-effectiveness studies have been performed concerning the addition of [^18^F]FET PET imaging when there is diagnostic uncertainty about pseudoprogression *versus* tumor progression in glioblastoma patients after concomitant chemoradiation.

Moreover, in spite of the high diagnostic accuracy, opinions of treating physicians are divided. Besides, [^18^F]FET PET is not reimbursed in several countries. Consequently, this leads to a subset of centers offering [^18^F]FET PET, where the treating physicians have positive opinions about [^18^F]FET PET and where budget is available. Demonstrating benefit of early, consistent use of [^18^F]FET PET in terms of patient outcomes and cost-effectiveness is therefore crucial to justify and ensure widespread availability for all patients with glioblastomas. Therefore, we aim to conduct a nation-wide prospective, randomized clinical trial to assess patient benefit and health economic impact of management based on the addition of [^18^F]FET PET to MRI, compared to only use of MRI, in glioblastoma patients with diagnostic uncertainty about tumor recurrence *versus* pseudoprogression after concomitant chemoradiation. Clinical management will be based on the imaging findings in both groups and will follow regular clinical guidelines for the imaging-based diagnosis.

### Objectives {7}

#### Primary objectives

To determine, in glioblastoma patients with diagnostic uncertainty between pseudoprogression and tumor progression on a follow-up MRI after concomitant chemoradiation (“index MRI”), whether the clinical management guided by an additional [^18^F]FET PET scan leads to fewer unnecessary interventions and better health-related quality of life after 12 weeks, compared with management based on MRI only.

#### Secondary objectives

To determine, in glioblastoma patients with diagnostic uncertainty between pseudoprogression and tumor progression on a follow-up MRI after concomitant chemoradiation (“index MRI”), whether clinical management guided by an additional [^18^F]FET PET scan, when compared to management based on MRI only, leads to:

- Cost-effective intervention
- Shorter time-to-diagnosis
- Changes in overall survival
- Lower number of unnecessary treatment cycles
- Improved HRQOL over time (continuous measure), measured at t=6 weeks and t=6 months.

### Trial design {8}

**Fig. 1:**
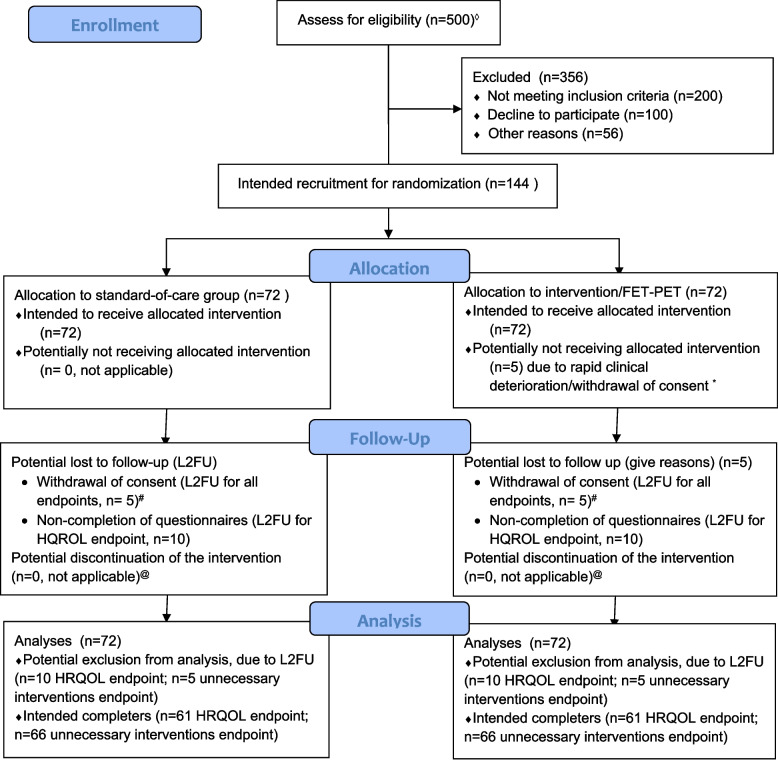
Study design flowchart. Potential number of patients, and number of patients meeting inclusion criteria, is based on real-life data from the included centers, very conservatively set at 50% of historical patient numbers 2019–2021. * Patients not receiving the intervention ([^18^F]FET PET) will be included in final analysis and not be replaced, according to an intention-to-treat design. Follow-up questionnaires will be administered if feasible. In this way, bias due to selective drop-out between arms will be avoided. ^#^Withdrawal of consent due to patient preference may occur, but is expected to be rare since patients have minimal extra burden during follow-up; transfer of care to a non-participating hospital is possible, but occurs only very infrequently in clinical practice. ^@^ The study features is a single-timepoint intervention (regular MRI, already available at baseline; versus [^18^F]FET PET), making discontinuation of the intervention during follow-up a non-relevant issue

This study is a prospective, national multicenter diagnostic randomized clinical trial, with a superiority design (lower proportion of unnecessary interventions and better HRQOL, compared with standard care). Patients that fulfill the inclusion criteria and who provide informed consent will be randomized in equal proportions between two arms (see Fig. 1):

- Arm A: investigational arm
  - Additional [^18^F]FET PET scan, as soon as possible after the index MRI
  - Clinical management is based on the index MRI and additional [^18^F]FET PET together
- Arm B: control arm
  - Clinical management is based on the index MRI only (standard care)

## Methods: participants, interventions and outcomes

### Study setting {9}

Patients are enrolled in 6 academic medical centers and 2 non-academic neuro-oncological hospitals in the Netherlands.

### Eligibility criteria {10}

#### Inclusion criteria

- Patients with a glioblastoma, IDH-wildtype, WHO grade 4 (according to WHO 2021 criteria), treated with standard-of-care temozolomide-based concomitant chemoradiation (60 Gy in 30 fractions or 40 Gy in 15 fractions) [17].
- Age ≥18 years.
- Follow-up MRI ≥ 3 months after the end of the temozolomide-based concomitant chemoradiation.
- New or increased enhancement within the high-dose radiation field (defined as within the 80% isodose line) on follow-up MRI.
- First moment of clinicoradiological uncertainty regarding the diagnosis (≥ 3 months after the end of concomitant chemoradiation): pseudoprogression or tumor progression. The determination of “uncertainty” is made by the treating physician, preferably in the multidisciplinary tumor board, based on available clinical and standard-of-care MRI-data, which generally includes perfusion-weighted MRI.
- Previous usage of bevacizumab as a symptom treatment is allowed. However, inclusion is only allowed at the first moment of clinical doubt between pseudoprogression and tumor progression, not at later timepoints.

#### Exclusion criteria

- Previous treatment for recurrence of disease.
- Largest diameter of the enhancing lesion of less than 1 cm on the index MRI, usually measured in the axial plane.
  In the newest RANO PET-criteria, it is advised to use [^18^F]FET PET for increasing lesions only in cases with a minimum lesion size [18].
- Life expectancy of less than 6 months, determined by the treating physician.
- Contra-indications for PET (claustrophobia, inability to lay still).
- Women of childbearing potential without adequate contraception during randomization and possible [^18^F]FET PET scan.
- Any other concomitant disease that may influence PET imaging or clinical outcomes of this study. This includes but is not limited to cerebral inflammatory diseases and other cancers with brain- or leptomeningeal metastases.

### Who will take informed consent? {26a}

When patients fulfill the in- and exclusion criteria, they will be asked by the treating physician if they are willing to be informed about the study. Subsequently, study information will be provided to the patient. Patients are given a 48-h informed consent window. Patients will be asked for informed consent by an authorized researcher.

### Additional consent provisions for collection and use of participant data and biological specimens {26b}

In the patient information letter, patients are asked for consent to use their pseudonymized data, including data on clinically performed tissue analyses (if available), in future studies and share their pseudonymized data with other neuro-oncologic research centers.

No biological specimens will be collected for the purpose of this study.

## Interventions

### Explanation for the choice of comparators {6b}

Clinical management based on MRI only is chosen as comparator as this is standard-of-care according to current Dutch- and international guidelines [19].

### Intervention description {11a}

Patients in the investigational arm will undergo the extra [^18^F]FET PET scan, with use of the O-(2-^18^F-fluoroethyl)-L-tyrosine ([^18^F] FET) tracer. The tracer will be provided free of charge by Curium™ for a subset of the patients (based on logistical availability and scheduling per patient). For this study, Curium™ does not have a role in the design, execution, analysis, interpretation, manuscript writing and/or decision to publish.

[^18^F]FET PET scanning will be performed according to the joint European Association of Nuclear Medicine (EANM)/European Association of Neurooncology (EANO)/Response Assessment in Neurooncology (RANO) guideline [20]. In most patients, a static scan (20–40 min post-injection) will be performed. Patients will be asked for additional (opt-in) consent to undergo a dynamic [^18^F]FET PET scan. If the logistics of the research site allow for this scan, these patients will undergo dynamic scanning (continuous scanning 0–40 min post-injection). Interpretation will be done by a nuclear medicine physician from the local center according to current European guidelines [20]. Central review will be performed by a panel of nuclear medicine physicians from the study team, specialized in [^18^F] FET PET scans.

Patients are treated according to current standard-of-care that is determined by the treating physician/multidisciplinary team, but will be based on the diagnosis from the static [^18^F]FET PET findings:

- In case of [^18^F]FET PET-diagnosis of tumor recurrence: a change in treatment regime will be made. Redo-debulking will be done only for clinical indications, including mass reduction or cytoreduction.
- In case of [^18^F]FET PET-diagnosis of pseudoprogression, or equivocal results: the ongoing treatment or observation will be continued, with addition of steroids and/or bevacizumab if needed for symptom relief. Redo-debulking will only be done for the rare indication of refractory symptoms of mass effect due to pseudoprogression.

### Criteria for discontinuing or modifying allocated interventions {11b}

This study has a single time-point intervention, making discontinuation of the intervention during follow-up a non-relevant issue. When a participant in the intervention group does not undergo the [^18^F]FET PET scan, this can be due to worsened clinical condition (or death) or participant request.

### Strategies to improve adherence to interventions {11c}

As the intervention is a single [^18^F]FET PET scan that is performed almost directly after randomization, no specific monitoring adherence procedures are applicable.

### Relevant concomitant care permitted or prohibited during the trial {11d}

Standard-of-care follow-up with MRI scans and standard-of-care diagnostic procedures and treatments such as biopsy, debulking, chemotherapy, reirradiation, and supportive therapies (e.g., bevacizumab, dexamethasone) are allowed in this trial. There is no prohibited concomitant care in this trial.

### Provisions for post-trial care {30}

As [^18^F]FET PET is a well-established, safe imaging method in glioma patients and is recommended in Dutch and international guidelines as a standard-of-care option, the risk of suffering harm from trial participation is considered negligible. Therefore, post-trial care provisions such as post-trial monitoring/visits are not applicable. Standard clinical follow-up of the participants will be performed by their treating physicians, ensuring sufficient care.

### Outcomes {12}

#### Primary study outcomes

##### Co-primary outcome 1

Undergoing any unnecessary intervention(s), as defined below. Determination of this dichotomous outcome measure “unnecessary interventions” will be done retrospectively, by the researchers and the treating physician after 6-month follow-up.

An “unnecessary intervention” is defined as:

- Biopsy or debulking for diagnostic uncertainty and/or with pseudoprogression as the final pathological diagnosis.
- The administration of one or more cycles of (temozolomide) chemotherapy as continued treatment for presumed pseudoprogression, in cases where the final diagnosis is tumor progression. The final diagnosis is based on clinicoradiological- and/or histopathological evidence.
- Starting and/or altering chemotherapy for presumed tumor progression, in cases where the final diagnosis is pseudoprogression.
- The administration of one or more doses of bevacizumab for presumed pseudoprogression, in cases with a final diagnosis of tumor progression.

##### Co-primary outcome 2

Health-related quality of life (HRQOL) (continuous measure): the European Organization for Research and Treatment of Cancer Quality of Life Core Questionnaire (EORTC-QLQ-C30) + Brain Cancer Module (EORTC-QLQ-BN20) at *t* = 12 weeks, preferably completed before follow-up imaging is performed.

#### Secondary outcomes

- Time-to-diagnosis (defined from the date of the index MRI result to the date of eventual management, including watchful waiting).
- Overall survival (defined from the date of the index MRI result to the date of death).
- Number of unnecessary treatment cycles during 6 months follow-up period (chemotherapy, bevacizumab).
- Cost-effectiveness, expressed in terms of incremental costs, incremental Quality-Adjusted Life Years (QALY), and Incremental Cost-Effectiveness Ratio (ICER). This is measured from a healthcare and societal perspective with use of three questionnaires: the iMTA Medical Consumption Questionnaire (iMCQ), the iMTA Productivity Cost Questionnaire (iPCQ), and EuroQol 5D-5L (EQ-5D) at *t* = 6 weeks, *t* = 12 weeks, and *t* = 6 months.
- HRQOL (continuous measure): EORTC-QLQ-C30 + BN20 at *t* = 6 weeks and *t* = 6 months (preferably before follow-up imaging is performed).
- PET characteristics as predictors of overall survival and progression-free survival outcomes.
- Determinants of unnecessary interventions.

#### Other study parameters

- Patient characteristics: sex, age.
- Oncological characteristics: date of diagnosis of initial tumor, O^6^-methylguanine DNA methyltransferase (MGMT) promotor methylation status, CDKN2A/B-status, Karnofsky performance score at baseline and during clinical examination moments.
- Questionnaires EORTC-QLQ-C30 + BN20, EQ-5D, iPCQ and iMCQ at baseline, measured between informed consent and randomization.
- Diagnostic- and treatment characteristics: surgical- and concomitant chemoradiation characteristics, usage of bevacizumab, dosage of dexamethasone, follow-up scans, blood tests.
- Final diagnosis during follow-up after study intervention: tumor recurrence or pseudoprogression.
- Notes from multidisciplinary meetings, describing diagnostic uncertainty and initial management after diagnostic imaging.

The schedule of assessments is illustrated in Table 1.

**Table 1:** Schedule of assessments

Visit	Index MRI result	Informed consent	Randomization	FET PET^a^	FET PET result^a^				
Study time	Day 1	± Days 3–14	±Days 5–16	± Days 7–30	± Days 10–33	Week 6	Week 12	Month 6	End of trial
Unnecessary interventions								X	
EORTC-QLQ-C30 + BN20/EQ-5D/iPCQ/iMCQ		X				X	X	X	
Karnofsky performance score	X				X^a^				
Final diagnosis								X	
Survival status								X	X^b^
Healthcare consumption								X	
Adverse events^c^				X	X				

^a^The intervention group only

^b^A survival sweep will be performed at the end of the trial

^c^All possibly/probably related adverse events will be reported for 30 days in both groups. All serious adverse events will be reported for 7 days in both groups

### Participant timeline {13}

**Fig. 2:**
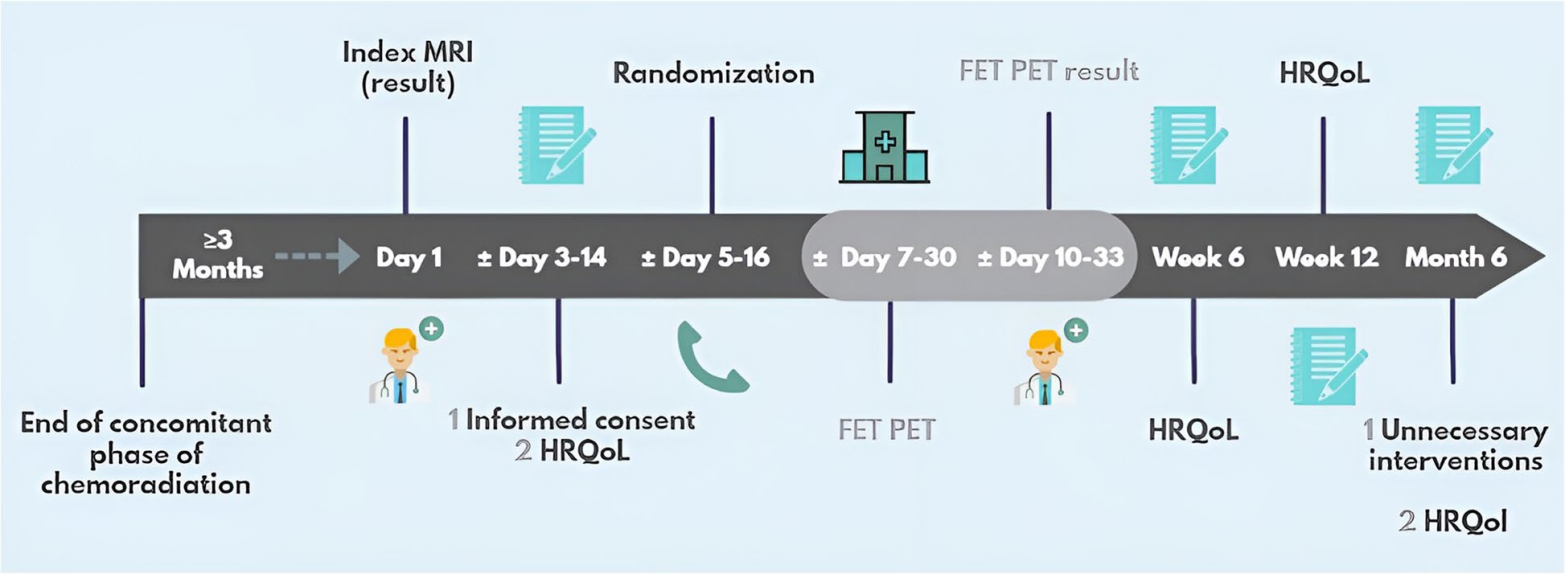
Participant timeline

Figure 2 shows the participant timeline.

### Sample size {14}

From current practice (survey among the participating centers), we estimate that 37% of the glioblastoma patients that meet our inclusion criteria, eventually undergo unnecessary interventions (control arm). In the intervention arm, we expect this proportion to be much lower (~ 12% from clinical scenarios), due to the high accuracy of [^18^F]FET PET. Using a two-sided *Z* test with pooled variance with an alpha of 0.05, based on a power of 90%, we need 61 patients in each arm, so 122 in total. Due to the intention-to-treat set-up and unnecessary treatment as outcome measure, we expect that virtually all patients can be included in final analysis, with minimal drop-out. For the co-primary endpoint of HRQOL (EORTC-QLQ-C30 + BN20) at *t* = 12 weeks, drop-out due to missing questionnaire data is estimated at 10%. With addition of this drop-out rate, we will randomize 144 patients, 72 patients per study arm.

Methodologically, the two primary endpoints are hierarchical: the first (unnecessary interventions) must be met before the second (HRQOL) is formally tested. Since HRQOL may be influenced by earlier diagnosis from FET PET, the primary endpoint of unnecessary interventions should be met first to obtain a HRQOL result which is not caused solely by such a timing effect. This justifies a fixed-sequence testing approach—each tested at *p* = 0.05. If the first test is not significant, HRQOL results will be considered exploratory. The sample size was powered for the first endpoint, with a 10% increase to account for potential missing HRQOL data; a formal power calculation for HRQOL was not feasible due to limited prior data.

### Recruitment {15}

Several strategies will be used to achieve adequate participant enrollment as well as completeness of data acquisition. Firstly, collaborating centers are encouraged to include eligible patients by informing them by an initiation visit, regular investigator meetings, a trial website, posters, a patient animation video and an information pocket card. Secondly, potential participants are encouraged to participate by providing them with comprehensible information in multiple ways: during the hospital visits, via a patient animation video, patient information letter and a short hand-out with images. This comprehensible information is crafted in collaboration with representatives of the “brain tumor contact group” of patient organization “Hersenletsel.nl.”

## Assignment of interventions: allocation

### Sequence generation {16a}

The computer-based randomization tool Ldot ® (by MEMIC, Maastricht University, https://www.ldot.nl/) will be used. Block randomization will be done with stratification by treatment center.

### Concealment mechanism {16b}

The computer-based randomization tool Ldot will be used, which will ensure study arm allocation is not known in advance by the researcher or treating physician.

### Implementation {16c}

Authorized researchers will enroll participants. The computer-based system Ldot will generate the allocation sequence and will assign participants to the interventions. The result of Ldot will be communicated to the participant by the authorized researcher.

## Assignment of interventions: blinding

### Who will be blinded {17a}

Due to the study design in which an additional [^18^F]FET PET scan will be performed, blinding is not possible.

### Procedure for unblinding if needed {17b}

Not applicable, the patients and researchers/physicians in this trial are not blinded.

## Data collection and management

### Plans for assessment and collection of outcomes {18a}

The data will prospectively be collected from medical charts, pathology reports, radiology reports, and web-based- and hardcopy questionnaires (EORTC-QLQ-C30 + BN20, EQ-5D, iPCQ, and iMCQ). Training of assessors of the different centers is performed by meetings. The EORTC-QLQ-C30 is a 30-item questionnaire designed to measure quality of life in all cancer patients [21]. The EORTC-QLQ-BN20 was developed as a site-specific supplement to the QLQ-C30 for use among brain cancer patients. The QLQ-BN20 consists of 20 questions translated into four summary scores and seven symptom scales [22, 23].

Cost-effectiveness of the study arms will be determined from a societal perspective. Life years, QALYs, and costs will be assessed. Life years and QALYs will be informed by the trial population and EQ-5D questionnaire. The EQ-5D is a 5-item scale consisting of mobility, self-care, usual activities, pain, and anxiety [24]. The EQ-5D is a generic utility questionnaire which is used in cost-effectiveness analyses to inform health technology assessment and allows for direct comparison between different diseases [25, 26]. Costs related to healthcare services by healthcare professionals (e.g., in- and out-patient visits), diagnostics (e.g., MRI scans, blood tests), and treatments (e.g., chemotherapy) will be extracted from electronic healthcare records from participating centers. The questionnaire iMCQ will be used to reveal the type and number of consultations by healthcare professionals in the primary, secondary, and domestic care [27]. Work-related costs, such as productivity loss, will be taken into account and measured by the iPCQ questionnaire, which is applicable to national and international studies [28].

[^18^F]FET PET imaging data will be gathered from the different centers as well. To ensure reliable and comparable [^18^F]FET PET data across all participating centers, a standardized scanning and review process is implemented through multiple ways. Firstly, the PET scanners of all participating centers will be harmonized. Secondly, training of local nuclear medicine physicians will be performed. Lastly, the [^18^F]FET PET scans performed at the individual centers will undergo local analysis, but will also undergo rapid central review by a team of independent nuclear medicine physicians specialized in [^18^F]FET PET scans. This is part of clinical practice as clinical decisions are based on these [^18^F]FET PET results. In case of discordance between local analysis and central review results, a consensus meeting will be held between one/multiple centralized reviewer(s) and the local nuclear medicine physician. The result of the central review will serve as the main outcome to guide clinical management.

### Plans to promote participant retention and complete follow-up {18b}

For the outcome measure “unnecessary treatments,” no, or minimal, missing data are expected, as data are all available in the electronic patient files. Dedicated study-specific data acquisition is performed by study personnel. Due to the organization of Dutch healthcare, loss to follow-up of patients is minimal.

For the health-related quality of life questionnaires, the participants will be contacted by secured e-mails or letters and asked to complete the set of questionnaires at each time point.

When the questionnaires are not completed after a certain period, the coordinating researcher will contact the patient to explore reasons for the delay and enhance motivation and participation.

### Data management {19}

Study sites will register a patient in the secured web-based application Ldot, which will generate a unique study registration (USR) number. All data collected for the study will be stored coded in electronic case report forms in Castor EDC ® (https://www.castoredc.com/). Online and hardcopy questionnaires will be sent to the patients by Ldot.

As Ldot and Castor will be connected, data of the online questionnaires will be sent to the Castor database in a coded format, without patient identifiers. Data of the hardcopy questionnaires will be entered in the Castor database by the coordinating researcher after secured login. The hardcopy questionnaires will then physically be stored in a secure archive of the UMC Utrecht. In order to perform the central review of the [^18^F]FET PET scans for clinical decisions, [^18^F]FET PET and accompanying MRI imaging data will be shared and stored with use of the Research Imaging Architecture (RIA), a fully secure data storage infrastructure of the UMC Utrecht. All data will be analyzed by the investigators of this study and the data will be stored for a maximum of 15 years.

### Confidentiality {27}

To register a patient in Ldot, each user will get a personal account to allow secured access to his own studies, site(s), and subjects. A “role based” access to the system will avoid unauthorized data access and prevents users to perform action they are not allowed to do. After registration of the patient in Ldot, this system will generate a unique study registration (USR) number. The unique study registration (USR) number and corresponding patient identifiers (name, date of birth, sex, date of inclusion and address) will be securely stored within the Ldot database.

The participants personal data will remain confidential and will not be published in any way; however, the sponsor’s monitor or representative and regulatory representatives (FDA and/or European Communities EU Notified Body Representatives), auditors, and inspectors may have access to medical files in order to verify authenticity of data collected. Upon informed consent, patients are aware that their patient identifiers will be used in order to send questionnaires via Ldot.

### Plans for collection, laboratory evaluation, and storage of biological specimens for genetic or molecular analysis in this trial/future use {33}

Not applicable, we will not collect biological specimens as part of this study.

## Statistical methods

### Statistical methods for primary and secondary outcomes {20a}

Results will be analyzed with use of an intention-to-treat analysis.

#### Primary study parameter(s)

1. Unnecessary interventions
  The “unnecessary interventions” outcome will be collected as a dichotomous outcome measure (yes/no), so the percentage of patients undergoing unnecessary interventions can be calculated for both groups. Then, a two-sided *Z*-test will be performed with pooled variance with an alpha of 0.05.
2. Health-related quality of life
  The primary outcome HRQOL will be captured and assessed as a continuous measure by EORTC QLQ-C30 and BN20 questionnaires through a timepoint analysis at *t* = 12 weeks. Compound scores will be calculated for each patient according to the standard scoring algorithms for these questionnaires [21, 23]. Group comparisons at this *t* = 12-week timepoint will be conducted using Student’s *t* test. Results will be interpreted in terms of statistically significant difference and clinical relevance between study arms. Statistical difference will be assessed via a two-sided *p*-value ≤ 0.05.

#### Secondary study parameter(s)

1. Time-to-diagnosis
  Time-to-diagnosis will be defined as the period from the date of the index MRI result to the date of the start of the final treatment. A Kaplan–Meier analysis will be performed. Groups will be compared with use of the log-rank test.
2. Overall survival
  Overall survival will be defined as the period from the date of the index MRI result to the date of death. A Kaplan–Meier analysis will be performed. Groups will be compared with use of the log-rank test.
3. Number of unnecessary treatment cycles
  The number of unnecessary treatment cycles of chemotherapy and bevacizumab will be calculated for both groups. The scores of both groups will be compared by means of the Mann–Whitney *U* test.
4.  Cost-effectiveness
  Cost-effectiveness of the study arms will be determined from a societal perspective. Life years, QALYs, and costs will be assessed (See “Plans for assessment and collection of outcomes”). Direct and indirect costs will be included in the analysis. If benefits and costs are expected to persist beyond trial time horizon, a decision analytic model will be built. If not, a trial-based economic evaluation will be conducted.
  The cost-effectiveness analysis will be conducted in adherence to the 2024 version of the Dutch guidelines for economic evaluations [25].
  Outcome of the cost-effectiveness analysis will be expressed as incremental Costs, incremental Quality-Adjusted Life Years (QALY), and Incremental Cost-Effectiveness Ratio (ICER). The results of the cost-effectiveness analysis will be combined with observed and extrapolated uptake to estimate the budget impact over time. The budget impact analysis (BIA) will be performed according to the Dutch guidelines and the BIA guideline (“BIA leidraad”) and BIA calculation tool will be utilized [29].
5. Health-related quality of life
  The secondary outcome HRQOL will be captured and assessed as a continuous measure by EORTC QLQ-C30 and BN20 questionnaires through timepoint analyses at *t* = 6 weeks and *t* = 6 months. Compound scores will be calculated for each patient according to the standard scoring algorithms for these questionnaires [21, 23]. Group comparisons at these timepoints will be conducted using Student’s *t* tests. Results will be interpreted in terms of statistically significant difference and clinical relevance between study arms. Statistical difference will be assessed via a two-sided *p*-value ≤ 0.05. Additionally, secondary HRQOL will be evaluated longitudinally as a continuous measure using a mixed model for repeated-measures analysis, examining changes in individual patients’ raw scores over time. Previous literature indicates HRQOL analyses become unreliable when the response rate falls below 60% of all participants [30]. Should this occur, data analysis will be limited to the timepoints that meet the 60% response criterion.
6. PET characteristics to predict clinical outcome
  In the intervention arm, we will perform exploratory analyses of the PET characteristics, both from static and (if available) dynamic PET images, such as SUV values and SUV ratio’s, as predictors of overall survival and progression-free survival, by cox analyses.
7. Other study parameters
  Lastly, determinants of unnecessary interventions will be investigated, including effect modifiers of the effect of [^18^F]FET PET on outcome. In order to answer this research question, a multivariable logistic regression analysis will be performed.

### Interim analyses {21b}

Not applicable, we will not perform an interim analysis as this could lead to inaccurate estimations of the intervention effect.

### Methods for additional analyses (e.g., subgroup analyses) {20b}

As treatment center will be used for stratification and it is expected that some of the centers will recruit small numbers of patients, no adjusting for this stratification factor will be performed in the primary analysis. However, a correction analysis for treatment center will be performed as sensitivity analysis. In addition, correction analyses for MGMT promoter methylation status (methylated versus unmethylated versus unknown), Karnofsky performance score (< 70 vs ≥ 70), and prior surgical procedure (biopsy versus resection) will be performed as sensitivity analyses [31].

### Methods in analysis to handle protocol non-adherence and any statistical methods to handle missing data {20c}

In case more than 10% of the patients in the intervention arm do not undergo the [^18^F]FET PET scan, an additional per-protocol analysis will be performed. This traditional per-protocol analysis will be supplemented by an instrumental variable analysis in which the complier average causal effect (CACE) will be estimated for the subpopulation of patients undergoing the extra [^18^F]FET PET scan. For the primary outcome measure of unnecessary interventions, no, or minimal missing data are expected, as data are all available in the electronic patient files. Dedicated study-specific data acquisition is performed by study personnel. Due to the organization of Dutch healthcare, loss to follow-up of patients is minimal. However, in the event that missing data do occur, imputation will not be applied to the primary outcome measure; (multiple) imputation is not considered to be optimally reliable or appropriate due to the nature of this outcome and its limited predictability. The occurrence of unnecessary treatments is highly dependent on contextual factors, such as physician decision-making and the clinical course of the illness, which makes accurate imputation difficult and potentially misleading. Therefore, for this specific outcome, an available-case analysis will be conducted including only those participants with available primary outcome data—expected to be all, or virtually all randomized patients. Where appropriate, sensitivity analyses will be performed to evaluate the robustness of the findings.

Regarding the HRQOL outcomes, standards of the field will be followed by using methods that are robust to missing data, e.g., linear mixed modeling combined with single-timepoint analyses.

### Plans to give access to the full protocol, participant-level data and statistical code {31c}

The full protocol and statistical code will be available upon request once the report has been published.

Participant-level data concerning baseline characteristics and major clinical outcomes will be made available as supplemental document of the article. Other participant-level data may be provided to non-commercial researchers upon reasonable request.

## Oversight and monitoring

### Composition of the coordinating center and trial steering committee {5d}

The main team of the coordinating center consists of two principle investigators and one coordinating researcher, who meet on a weekly basis to discuss day-to-day issues but also the implementation and management of the trial. The two principle investigators, the trial agency of the division and the project controller form the basis of the steering committee. They monitor the trial progress and provide oversight. The principle investigators of the local centers coordinate the execution of the trial in their center and together with the steering committee they form the study group. An unaffiliated physician is accessible for independent patient support. In addition, a datamanager is involved in overseeing data collection, storage, analysis, and quality control processes within the research project.

The monitoring of the study in all centers will be performed by the trial agency of the sponsor.

### Composition of the data monitoring committee, its role and reporting structure {21a}

As [^18^F]FET PET is a well-established, safe imaging method in glioma patients and is recommended as an optional imaging modality for these patients in Dutch and international guidelines, the risk of participation in this trial is considered negligible. Due to this negligible risk, establishing a data monitoring committee is not needed, according to the NFU (Nederlandse Federatie van Universitaire Medische Centra) guidelines [32].

### Adverse event reporting and harms {22}

All possibly/probably related adverse events (AEs) reported spontaneously by the subject or observed by the investigator or his staff will be recorded. These possibly/probably related AEs will be reported for 30 days in both groups. All serious adverse events (SAEs) will be systematically reported for 7 days in both groups, regardless of relatedness. Both time windows start from the date of the PET scan in the intervention group and 7 days after randomization in the control group. The risk of (S)AEs caused by the additional [^18^F]FET PET scan is considered to be negligible. However, due to the poor life expectancy of glioblastoma patients, expected SAEs are hospitalization, persistent or significant disability or incapacity, or death due to glioblastoma progression. All AEs and SAEs meeting the aforementioned criteria will be included in trial publications. CTCAE 5.0 terminology will be used for classification [33].

### Frequency and plans for auditing trial conduct {23}

Independent regulatory authorities are permitted access to the data and study documentation essential for control purposes.

### Plans for communicating important protocol amendments to relevant parties (e.g., trial participants, ethical committees) {25}

Important protocol modifications such as changes to study objectives, design, eligibility criteria, outcomes, and analysis will be reported to the Medical Ethics Review Committee “NedMec.” Moreover, the ClinicalTrials.gov registration will be revised in response to any modifications.

### Dissemination plans {31a}

Results will be published in a peer-reviewed medical journal with open access, unreservedly, and regardless of their nature, in accordance with the Central Committee on Research Involving Human Subjects (CCMO) statement on publication policy. Every center that includes patients will be provided authorship, provided that the author(s) meet the ICMJE requirements for authorship (www.icmje.org). Additionally, results will be presented at national and international scientific gatherings and distributed among patient advocacy groups including the “brain tumor contact group” of patient organization “Hersenletsel.nl.”

## Discussion

The FET POPPING study aims to assess the clinical and health economic impact of the addition of a [^18^F]FET PET scan during diagnostic uncertainty in the follow-up of glioblastoma patients who received concomitant chemoradiation in a randomized clinical trial. Our hypothesis is that the addition of a [^18^F]FET PET scan leads to a faster accurate diagnosis, which will lead to fewer unnecessary interventions, a better health-related quality of life, and a net decrease in healthcare costs.

Literature concerning the clinical- and health economic impact of management based on an additional [^18^F]FET PET scan during diagnostic uncertainty in the follow-up of adult glioblastoma patients is scarce. One retrospective study evaluated the clinical impact of a combined [^18^F]FET PET/MRI performed when progressive disease was suspected during or after post-operative therapy in a mixed group of brain tumor patients. This study found that in a subgroup of IDH-wildtype high-grade glioma patients, the combined [^18^F]FET PET/MRI changed the clinical management in 47% [34]. One prospective study evaluated the clinical impact of a combined [^18^F] FET PET/MRI when there was diagnostic uncertainty on follow-up MRI in patients with grade 3/4 glioma and brain metastases. This trial showed that the hybrid [^18^F]FET PET/MRI positively impacted the routine clinical care [6]. In an ongoing prospective clinical trial, of which recruitment of patients is expected to be completed in 2024, a [^18^F]FET PET will be performed in newly diagnosed glioblastoma patients at up to three time points, among others within 14 days of suspected clinical and/or radiological progression on MRI [35].

A key advantage of the FET POPPING trial is its prospective randomized clinical design, which yields the highest level of evidence. In addition, if our hypothesis proves to be accurate, the [^18^F]FET PET scan could be reimbursed by the health insurance providers, facilitating its widespread availability across all neuro-oncology centers in the Netherlands.

In summary, the FET POPPING trial will assess the clinical- and health economic impact of the addition of a [^18^F]FET PET scan during diagnostic uncertainty in the follow-up of glioblastoma patients who received concomitant chemoradiation. Important endpoints are the percentage of unnecessary interventions, health-related quality of life, and healthcare costs.

## Trial status

The current protocol is version 4.0, date 16–05–2025. The study was officially initiated in August 2024 and recruitment of participants began in January 2025. The recruitment is estimated to end in December 2027.

## Data Availability

Pseudonymized data may be shared with participating hospitals and/or other neuro-oncologic centers for the purpose of conducting neuro-oncological research. These data will remain the property of this project and will only be disclosed upon request and after a quality review by the project’s research board. The trial is registered at ClinicalTrials.gov (NCT06480721) and results information will be submitted to ClinicalTrials.gov.

## Abbreviations

BIA: Budget impact analysis
CACE: Complier average causal effect
CCMO: Central Committee on Research Involving Human Subjects; in Dutch: Centrale Commissie Mensgebonden Onderzoek
EORTC-QLQ-BN20: European Organization for Research and Treatment of Cancer Quality of Life Brain Cancer Module
EORTC-QLQ-C30: European Organization for Research and Treatment of Cancer Quality of Life Core Questionnaire
EQ-5D: EuroQol 5D-5L
FET: Fluoro-ethyl-tyrosine
HRQOL: Health-related quality of life
ICER: Incremental Cost-Effectiveness Ratio
IDH: Isocitrate dehydrogenase
iMCQ: iMTA Medical Consumption Questionnaire
iPCQ: iMTA Productivity Cost Questionnaire
L2FU: Lost to follow-up
MGMT: O^6^Methylguanine DNA methyltransferase
MRI: Magnetic resonance imaging
NFU: Nederlandse Federatie van Universitaire Medische Centra
PET: Positron emission tomography
QALY: Quality-Adjusted Life Years
RANO: Response Assessment in Neuro-Oncology working group
WHO: World Health Organization
